# TLR3/TRIF signalling pathway regulates IL-32 and IFN-β secretion through activation of RIP-1 and TRAF in the human cornea

**DOI:** 10.1111/jcmm.12495

**Published:** 2015-03-06

**Authors:** Ga Bin Park, Dae Young Hur, Yeong Seok Kim, Hyun-Kyung Lee, Jae Wook Yang, Daejin Kim

**Affiliations:** a Department of Anatomy, Inje University College of MedicineBusan, Korea; b Ocular Neovascular disease Research Center, Inje University Busan Paik HospitalBusan, Korea; c Department of Internal Medicine, Inje University Busan Paik HospitalBusan, Korea; d Department of Ophthalmology, Inje University Busan Paik HospitalBusan, Korea

**Keywords:** human cornea epithelial cells, Epstein–Barr virus, TRIF, RIG-I, RIP-1, IL-32

## Abstract

Toll-like receptor-3 (TLR3) and RNA helicase retinoic-acid-inducible protein-1 (RIG-I) serve as cytoplasmic sensors for viral RNA components. In this study, we investigated how the TLR3 and RIG-I signalling pathway was stimulated by viral infection to produce interleukin (IL)-32-mediated pro-inflammatory cytokines and type I interferon in the corneal epithelium using Epstein–Barr virus (EBV)-infected human cornea epithelial cells (HCECs/EBV) as a model of viral keratitis. Increased TLR3 and RIG-I that are responded to EBV-encoded RNA 1 and 2 (EBER1 and EBER2) induced the secretion of IL-32-mediated pro-inflammatory cytokines and IFN-β through up-regulation of TRIF/TRAF family proteins or RIP-1. TRIF silencing or TLR3 inhibitors more efficiently inhibited sequential phosphorylation of TAK1, TBK1, NF-κB and IRFs to produce pro-inflammatory cytokines and IFN-β than RIG-I-siRNA transfection in HCECs/EBV. Blockade of RIP-1, which connects the TLR3 and RIG-I pathways, significantly blocked the TLR3/TRIF-mediated and RIG-I-mediated pro-inflammatory cytokines and IFN-β production in HCECs/EBV. These findings demonstrate that TLR3/TRIF-dependent signalling pathway against viral RNA might be a main target to control inflammation and anti-viral responses in the ocular surface.

## Introduction

Toll-like receptors (TLRs) activate multiple inflammatory pathways and coordinate systemic defence against microbial pathogens. The improper activation of TLR pathways by endogenous or exogenous ligands may initiate tissue injury 1. Unlike TLR-7, -8 and -9, TLR3 signalling is MyD88-independent and utilizes adaptor protein toll/interleukin-1 receptor (TIR) domain-containing adaptor inducing IFN-β (TRIF) 2. TRIF also recruits additional proteins necessary for downstream signalling, including receptor-interacting protein-1 (RIP-1), tumour necrosis factor receptor-associated factor 6 (TRAF6) and TRAF-family member associated NF-κB-activator (TANK) binding kinase 1 (TBK1) 3.

The corneal epithelium serves as a barrier to protect the eye from external agents while maintaining corneal transparency 4–6. Corneal epithelial disorders are associated with various ocular surface diseases involving inflammation, a process that might disrupt corneal structure and vision changes 7. Corneal epithelium and inflammatory cells are involved in various pathologic conditions, including injury, repair and wound healing processes 8. The formation of pro-inflammtory cytokines and chemokines, including interleukin (IL)-1β, IL-6, IL-8, Monocyte chemoattractant protein-1 (MCP-1), tumour necrosis factor (TNF)-α, aggravates inflammation and tissue injury 9. Therefore, regulation of pro-inflammatory cytokines is one of the key processes to attenuate the corneal injury after infection. TLR2, TLR4 and TLR9 are expressed in the cornea and mediate corneal inflammation through the MyD88 adaptor molecule 10,11. TLR3 is also expressed within the corneal epithelium 12. The expressions of pro-inflammatory cytokines and IFN-β are up-regulated in HCECs exposed to herpes simplex virus-1 (HSV-1) 13. However, it is still unclear how TLR3 signalling and related pro-inflammatory cytokines are regulated in the ocular surface mucosa.

Interleukin-32 is a recently described cytokine produced by T lymphocytes, natural killer cells, epithelial cells, mast cells, keratinocytes and blood monocytes 14,15. IL-32 secretion is modulated by signals transferred from specific TLRs, such as TLR2, TLR3, TLR4, TLR5 and TLR6, in corneal epithelium 16. IL-32 also induces the production of pro-inflammatory cytokines, such as TNF-α, IL-1β, IL-6 and IL-8 by activating NF-κB and p38 mitogen-activated protein kinase (MAPK) 17,18. Ocular inflammation can lead to increased corneal epithelial permeability as a result of disrupted tight junctions (TJs). The components of TJs, including zonula occludens (ZO-1, ZO-2 and ZO-3) and adherens junctions (AJs) proteins (E-cadherin and β-catenin) are disappeared in corneal epithelium after inflammation 19. TNF-α, a pro-inflammatory cytokine 20, contributes to the ocular inflammation associated with infection 21, injury 22 and dry eye 23. Inhibition of TNF-α is effective in suppressing tissue inflammation, and the use of anti-TNF agents has emerged as a potential therapeutic modality in various autoimmune conditions, including Sjogren's syndrome 24. Conversely, lack of TNF-α potentiates pathogenic excess inflammation, fibrogenic response and neovascularization in alkali-burned mouse corneas 25. Further studies are needed to determine whether anti-TNF-α strategies are effective in treating ocular surface inflammation.

Epstein–Barr virus (EBV), also called human herpesvirus 4 (HHV-4), is one of the most common viruses in humans. EBV can infect a number of different cell types, including B cells and epithelial cells.

The herpes viruses including HSV-1, varicella zoster virus (VZV) and cytomegalovirus (CMV) are the common cause of viral keratitis that leads to corneal neovascularization 26. EBV is also detected in patients underwent cornea transplantation and viral keratitis 27. EBV infection of human B lymphocytes often leads to latency, a state in which most viral genes are not expressed 28. However, EBV-encoded small RNAs (EBERs), the most abundant small non-coding RNAs, are detected in human cells infected with EBV 29. Epithelial cells are a dominant and widespread source of pro-inflammatory cytokine IL-32 30. However, it is still unclear how IL-32 induces other inflammatory cytokines after corneal viral infection and what signalling pathways are involved in secretion of anti-viral type I interferon. We established EBV-infected human cornea epithelial cells (HCECs/EBV) as a model of virus-associated keratitis, which caused acute cornea necrosis and neo-vascularization in a previous study 31. This study investigates the differential signalling pathway of viral RNA component through TLR3 or RIG-I for pro-inflammatory cytokine secretion and IFN-β production using HCECs/EBV.

## Materials and methods

### Cell culture and reagents

Human cornea epithelial cells, human corneal epithelial cells, were purchased from Invitrogen-Gibco (Carlsbad, CA, USA). Cells were maintained in keratinocyte serum-free medium supplemented with Bovine Pituitary Extract (BPE, Invitrogen-Gibco) and human recombinant epidermal growth factor (EGF, Invitrogen-Gibco) at 37°C in 5% CO_2_. To establish EBV-infected HCECs, HCECs were added to cell-free EBV virions obtained from a B95-8 cell line (ATCC, Manassas, VA, USA), as previously described 31. The cultures were incubated for 1 day to 4 weeks. At 4 weeks after infection with EBV, EBV infection was confirmed by RT-PCR and Western blot to detect viral transcripts and proteins. HCECs/EBV in all experiments were used after verification of EBV infection. TLR3/dsRNA complex inhibitor was purchased from Calbiochem (San Diego, CA, USA). Bay 11-7082 and Necrostatin-1 were purchased from Selleckchem (Houston, TX, USA). BX795 was purchased from InvivoGen (San Diego, CA, USA).

### Quantitative real-time PCR

Total cellular RNA was extracted using an RNeasy Mini kit (Qiagen, Hilden, Germany) according to the manufacturer's protocol. cDNA was produced from 2 μg total RNA using oligo (dT) (Bioneer, Daejeon, Korea) and reverse transcriptase (Bioneer). Quantitative mRNA levels were measured using an ECO real-time PCR system (Illumina, Inc., San Diego, CA, USA) and a SYBR Green Master Mix kit (Takara, Tokyo, Japan) with specific primer sets (Table1). Relative mRNA quantification was calculated using the arithmetic formula 2^−▵▵Cq^, where ▵Cq is the difference between the threshold cycle of a given target cDNA and an endogenous reference cDNA.

**Table 1:** Sequences of oligonucleotide primers used for real-time PCR

Target	Primers (5′→3′)	
	Sense	Antisense
EBER1	AGG ACC TAC GCT GCC CTA GA	AAA ACA TGC GGA CCA
EBER2	GGA CAG CCG TTG CCC TAG TGG TTT CGG A	AAA ACA GCG GAC AAG CCG AAT ACC
TLR3	TCA CTT GCT CAT TCT CCC TT	GAC CTC TCC ATT CCT GGC
IL-32	GCC TTG GCT CT TGA ACT TTT G	CCG CCA CTG CTG TCT CCA GGT AG
IL-32α	CTG AAG GCC CGA ATG CAC CA	CCG TAG GAC TTG TCA CAA AA
IL-32β	CTG AAG GCC CGA ATG CAC CAG	GCA AAG GTG GTG TCA GTA TC
IL-32γ	TGA CAT GAA GAA GCT GAA GGC	CAT GAC CTT GTC ACA AAA GCT C
IL-32δ	TCT CTG ATG ACA TGA AGA AGC T	GCA AAG GTG GTG TCA GTA TC
IFN-β	GAT TCA TCG AGC ACT GGC TGG	CTT CAG GTA ATG CAG AAT CC
RIG-I	GCA TAT TGA CTG GAC GTG GCA	CAG TCA TGG CTG CAG TTC TGT C
RIP-1	GGG AAG GTG TCT CTG TGT TTC	CCT CGT TGT GCT CAA TGC AG
TRIF	GCC AGC AAC TTG GAA ATC AGC	GGG GTC GTC ACA GAG CTT G
TRAF1	TCCTGTGGAAGATCACCAATGT	GCAGGCACAACTTGTAGCC
TRAF2	TCCCTGGAGTTGCTACAGC	AGGCGGAGCACAGGTACTT
TRAF3	TCTTGAGGAAAGACCTGCGAG	GCGATCATCGGAACCTGAC
TRAF6	TTG CCA TGA AAA GAT GCA GAG G	AGC CTG GGC CAA CAT TCT C
TAK1	ATT GTA GAG CTT CGG CAG TTA TC	CTG TAA ACA CCA ACT CAT TGC G
TBK1	TGG GTG GAA TGA ATC ATC TAC GA	GCT GCA CCA AAA TCT GTG AGT
IRF3	CAC AGC AGG AGG ATT TCG G	CCT GGG TAT CAG AAG TAC
IRF7	GCT GGA CGT GAC CAT CAT GTA	GGG CCG TAT AGG AAC GTG C
β-actin	ATC CAC GAA ACT ACC TTC AA	ATC CAC ACG GAG TAC TTG C

### Immunoblotting

Cells were harvested and lysed in NP-40 buffer (Elpis Biotech, Daejeon, Korea) supplemented with a protease inhibitor cocktail (Sigma-Aldrich, St. Louis, MO, USA). To address phosphorylation events, an additional set of phosphatase inhibitors (Cocktail II, Sigma-Aldrich) was added to the NP-40 buffer. Protein concentration was determined using a BCA assay kit (Pierce, Rockford, IL, USA). The same volume of 2× Laemmli sample buffer (Elpis Biotech) was added to each lysate, and each protein sample (10 μg/sample) was immediately boiled for 5 min. at 100°C. Insoluble material was spun down at 16,000g. Total cell lysates (5 × 10^6^ cells/sample) were subjected to SDS-PAGE on a gel containing 15% (w/v) acrylamide under reducing conditions. Separated proteins were transferred to nitrocellulose membranes (Millipore Corp., Billerica, MA, USA). The membranes were blocked with 5% skim milk and commercial Western blot analysis was performed. Chemiluminescence was detected using an ECL kit (Advansta Corp., Menlo Park, CA, USA) and the multiple Gel DOC system (Fujifilm, Tokyo, Japan). The following primary Abs were used: E-cadherin, N-cadherin, β-catenin, Vimentin, ZO-1, TLR3, TRIF, RIP-1, RIG-I, TRAF1, TRAF2, TRAF3, TRAF6, phospho-TAK1 (Thr^184/187^), TAK1, phospho-TBK1 (Ser^172^), TBK1, phospho-IRF3 (Ser^396^), IRF3, phospho-IRF7 (Ser^471/472^), IRF7, phospho-p65 (Ser^536^), p65, p105/p50, p100/p52, Rel-B, PARP and β-actin were from Cell Signaling Technology (Beverly, MA, USA); β-tubulin was from BD Biosciences (San Diego, CA, USA).

### ELISA

At 24 or 48 hrs, the conditioned media was collected and the amount of IL-6, IL-8, VEGF, TNF-α and MCP-1 secreted by EBV-infected HCECs was quantified by a Single Cytokine ELISA Assay Kit (R&D systems, Minneapolis, MN, USA), according to the manufacturer's instructions. IL-32α was quantified by a Single Cytokine ELISA Assay Kit (BioLegend, San Diego, CA, USA). IL-32γ was quantified by the Single Cytokine ELISA Assay Kit (YbdY, Gwanlin, Korea). IFN-β was quantified by Single Cytokine ELISA Assay Kit (PBL Interferon Source, Piscataway, NJ, USA). Data are expressed as an average of the number of biological replicates ± SD.

### Detection of NF-κB, IRF3 and IRF7 translocation by fractionation

Mitochondrial and cytosol cellular fractions were prepared using a Cytosol/Mitochondria Fractionation Kit (Calbiochem, San Diego, CA, USA). Centrifugation at 600 × g for 5 min. was used to harvest 5 × 10^6^ cells with or without several treatments at 4°C. Cells were then washed twice with cold PBS. Afterwards, the cells were re-suspended in 250 μl cytosol extraction buffer containing a protease inhibitor cocktail and 1 mM dithiothreitol (DTT). After incubation on ice for 10 min., the cells were homogenized on ice using a Dounce tissue homogenizer. Homogenized cells were centrifuged at 700 × g for 10 min. at 4°C and supernatants were collected. Supernatants were then centrifuged again at 10,000 × g for 30 min. at 4°C. The resulting supernatants were harvested and designated as cytosolic fractions. The pellets were re-suspended in 50 μl mitochondria extraction buffer containing protease inhibitor cocktail and 1 mM DTT. The resulting samples were designated as mitochondrial fractions. Nuclear cellular fractions were prepared using a Nuclear/Cytosol Fractionation Kit (Biovision, Mountain View, CA, USA). Then, 2 × 10^6^ cells with or without various treatments were harvested and suspended in 200 μl cytosol extraction buffer A. After incubation on ice for 10 min., cell suspension was added with cytosol extraction buffer B followed by incubation on ice for 1 min. The obtained pellets were re-suspended in 100 μl nuclear extraction buffer mix and designated as nuclear fractions.

### Measurement of NF-κB activity by NF-κB DNA-binding ELISA assay

ELISA and a NF-κB p50/p65 Transcription Factor Assay Kit (Abcam, Cambridge, MA, USA) were used according to the manufacturer's protocol to quantify the DNA-binding activity of NF-κB. Briefly, nuclear extracts were transferred to a 96-well plate coated with a specific dsDNA sequence containing the NF-κB response element. NF-κB proteins bound to the target sequence were detected with a primary antibody and an HRP-conjugated secondary antibody. The absorbance was measured at 450 nm as a relative measure of protein-bound NF-κB. All fractions were stored at −80°C until further use.

### RNA-binding protein immunoprecipitation assay

As described previously 32, the RNA-binding protein immunoprecipitation assay was performed with a Magna RIP™ RNA-Binding Protein Immunoprecipitation Kit (Millipore) according to the manufacturer's instructions. Briefly, HCECs or EBV-infected HCECs at 80–90% confluence were lysed in RIP lysis buffer on ice after being washed in PBS and were then stored at −80°C until further use. Magnetic beads were prepared by initial PBS washes followed by incubation at room temperature for 30 min. with primary antibody raised against TLR3 (5 μg of total antibody used per immunoprecipitation). Extensive washes were performed prior to incubation of absorbed magnetic beads with previously collected cell lysates. Incubation of conjugated beads with lysate took place overnight at 4°C with rotation. The beads were thoroughly washed and digested with proteinase K (30 min. at 55°C) to disengage TLR3 containing ribonucleoprotein (RNPs) complexes. RNA from immunopurified RNPs were harvested *via* canonical phenol chloroform isoamyl extraction and further precipitated *via* ethanol. Immunoprecipitated RNA from TLR3-RNPs was then subjected to cDNA synthesis and qPCR analysis.

### Small interfering RNA transfection

Experimentally verified human TRIF-small interfering RNA (siRNA) duplex, RIG-I-siRNA duplex, and negative control-siRNA were obtained from Bioneer. Cells were seeded at a concentration of 1 × 10^5^ per well in a T75 flask and grown overnight. Cells in each T75 flask were then transfected with 200 nM siRNAs using Lipofectamine RNAiMAX Reagent (Invitrogen) according to the manufacturer's instructions. Cells were used for further experiments at 48 hrs after transfection.

### Statistical analysis

Data were expressed as mean ± SD. Statistical analysis was conducted using one-way anova. *P* < 0.05 were considered statistically significant.

## Results

### EBER1 and EBER2 are expressed and bound to TLR3 for IL-32 production in mesenchymal-like HCECs/EBV

**Figure 1:**
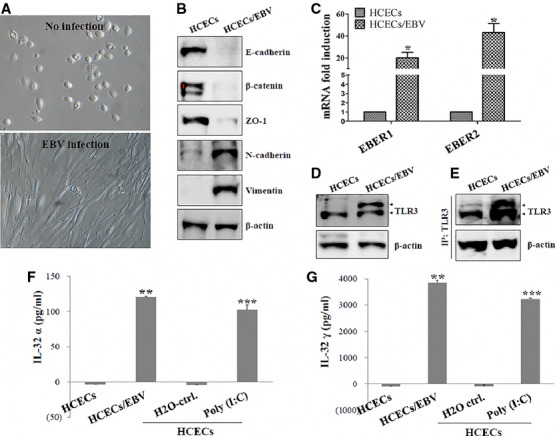
EBER1 and EBER2 are expressed and bind to TLR3 in EBV-infected HCECs. (A) Morphology of EBV-infected *versus* uninfected HCECs. Non-exposed HCECs had a typical cobblestone-like monolayer appearance (upper) while EBV infection (>4 weeks) induced phenotypic transition from cuboidal clustered epithelial cells to elongated fibroblast-like spindle-shaped cells with decreased cell-to-cell contact (lower). Morphology was observed under an inverted phase-contrast microscope. Photographs were taken at ×100 magnification by a digital camera. (B) Western blot analysis of EMT markers (E-cadherin, β-catenin, ZO-1, N-cadherin and Vimentin). (C–E) Analysis of EBER and TLR3 binding using RIP assay as described in the Materials and Methods section. (C) mRNA levels of EBER1 and EBER2 expression in EBV-infected and uninfected HCECs measured using real-time PCR. *P < 0.001 (HCECs *versus* HCECs/EBV). (D) Western blot of TLR3 expression in EBV-infected and uninfected HCECs. (E) After RNA-binding Protein Immunoprecipitation assay, TLR3 levels that binds to EBER1 and EBER2 in EBV-infected and uninfected HCECs were measured using immunoprecipitation. Quantitative levels of secretion by IL-32α (F) and IL-32γ (G) ELISA. Poly (I:C) (Sigma-Aldrich)-treated HCECs (10 μg/ml Poly (I:C) for 48 hrs) were used as a positive control for IL-32α and IL-32γ. **P < 0.001 (HCECs *versus* HCECs/EBV); ***P < 0.001 (H_2_O control *versus* Poly (I:C) treatment). Data are presented as the mean of three independent experiments, and error bars represent SDs of the means. Results are representative of three independent experiments.

First, we established a stable human viral keratitis model to study the signalling pathways of inflammation against viral RNA component in cornea epithelium. A loss of AJ proteins (E-cadherin, β-catenin) and ZO-1 after EBV infection while a high level of N-cadherin and vimentin were detected in HCECs/EBV compared to non-infected HCECs. Morphological changes in HCECs from ovoidal to spindle shaped after EBV infection were also observed (Fig.1A and 1B). The stability of EBER1 and EBER2 expression at 4 weeks after EBV infection in established HCECs/EBV was observed. EBER1 and EBER2 transcription was confirmed by quantitative real-time PCR. In addition, EBER1 and EBER2 expression was higher than that of EBV-transformed B cells (Fig. S1A). To examine whether EBER1 and EBER2 bind to TLR3, which is expressed intracellular HCECs/EBV, lysate of HCECs/EBV was collected for detecting target protein of EBER1 and EBER2 with RNA-binding protein immunoprecipitation assay. From purified RNA of TLR3-RNP complex, higher levels of EBER1 and EBER2 were detected with quantitative real-time PCR in HCECs/EBV (Fig.1C). TLR3 expression of HCECs/EBV was also up-regulated in total cell lysate and RNP complex compared to that of HCECs (Fig.1D and 1E). A TLR3-mediated signal regulates IL-32 secretion, and IL-32 induces the production of pro-inflammatory cytokines 16,17. The EBERs-TLR3 interaction was examined to investigate its effect on IL-32 production in HCECs/EBV. Compared to non-infected HCECs, total IL-32 and IL-32 isoforms (α, β, γ, and δ) were increased in mRNA expression in HCECs/EBV (Fig. S1B). IL-32α and IL-32γ secretion were also up-regulated in HCECs/EBV, even after stimulation with synthetic dsRNA, poly (I:C), in HCECs (Fig.1F and G). These data suggest that HCECs/EBV might be used as a model of human viral keratitis and RNA components of EBV stably bind to TLR3 to induce IL-32 production in HCECs/EBV.

### TRAFs/TAK/TBK1 signalling and NF-κB activation are promoted after elevation of TRIF, RIG-I, and RIP-1 in HCECs/EBV

**Figure 2:**
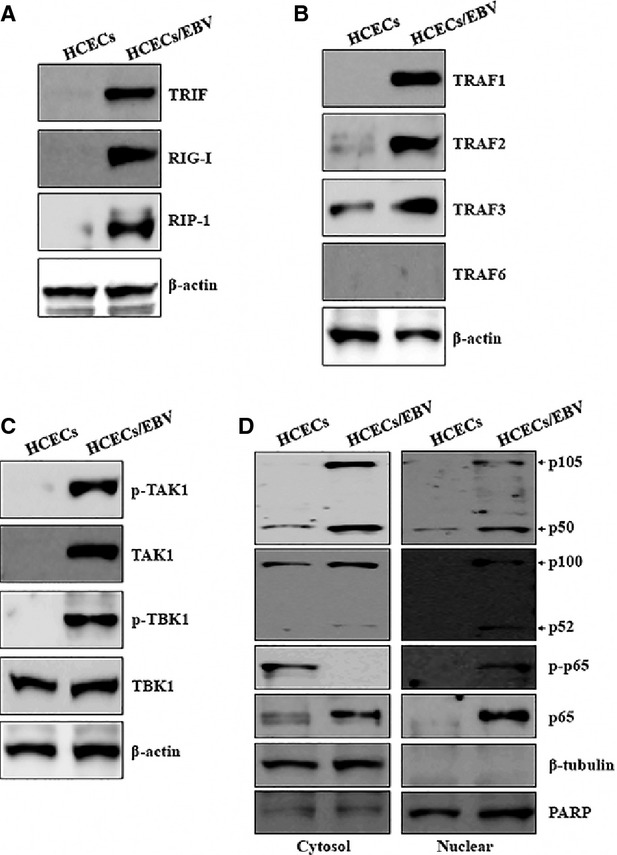
EBV induces expression of TRAF/TAK/TBK1 signalling and NF-κB activation in HCECs. (A–C) Total proteins were extracted from cell lysates and Western blots were performed with the following antibodies; (A) TRIF, RIG-I, RIP-1; (B) TRAF1, TRAF2, TRAF3, TRAF6; (C) phosphor-TAK1, TAK1, phosphor-TBK1, TBK1. β-actin served as an internal control. (D) Cytosolic extracts (left panel) or nuclear extracts (right panel) were analysed by Western blot using Abs against p105/p50, p100/p52, phospho-p65, and p65. A nuclear marker, PARP, and a cytosol marker, β-tubulin, were used to verify the purity of each fraction. Fractionation was performed as described in Materials and Methods. Results are representative of three independent experiments.

Next, the signalling pathway that induces IL-32 production was investigated in HCECs/EBV. RIG-I and TLR3 sense dsRNA and a replication intermediate for RNA viruses 33 to activate NF-κB 34. TRAF-family proteins connect TLR3 signals to transforming growth factor-β (TGF-β)-activated kinase 1 (TAK1), which plays a key role in the production of TNF-α and other inflammatory mediators by activating several MAPKs and NF-κB in B lymphocytes 35. Although TRAF6 mRNA did not change significantly, the expression level of other mRNAs, including TRAF1, TRAF2 and TRAF3, related to TLR3 and RIG-I signalling was increased in HCECs/EBV (Fig. S2). TRIF, a major adaptor protein of TLR3, was up-regulated as well as RIG-I. RIP-1, major protein that interacts with RIG-I, was expressed higher in HCECs/EBV than that of HCECs (Fig.2A). TRAF-family proteins (TRAF1 to 3) were also up-regulated in protein level, except for TRAF6 in HCECs/EBV (Fig.2B). TAK1 protein was induced, and phosphorylation of TAK1 and TBK1 adaptor proteins was observed in HCECs/EBV (Fig.2C). After EBV infection in HCECs, the total NF-κB protein level and nuclear levels of active NF-κB subunits p50 and p52 increased. NF-κB p65 and phosphorylated p65 were up-regulated and translocated to the nucleus in HCECs/EBV (Fig.2D). These data suggest that the TRAFs/TAK1/TBK1 activation might be involved in NF-κB activation and subsequent nuclear translocation for IL-32 production after viral infection in corneal epithelium.

### HCECs/EBV produces IFN-β through enhanced phosphorylation and nuclear accumulation of IRF3/IRF7

**Figure 3:**
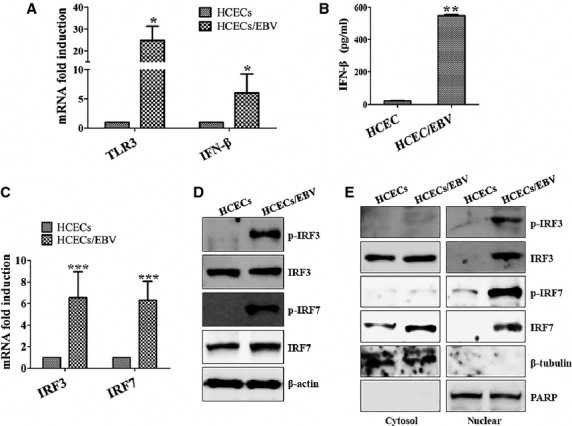
EBV produces IFN-β through enhanced phosphorylation and nuclear accumulation of IRF3/IRF7 in HCECs. (A) mRNA levels of TLR3 and IFN-β expression in EBV-infected and uninfected HCECs were measured using real-time PCR. *P < 0.001 (HCECs *versus* HCECs/EBV). (B) Quantitative levels of secretion by IFN-β ELISA. **P < 0.001 (HCECs *versus* HCECs/EBV). (C) Total RNA was extracted from cell lysates and real-time PCR was performed for IRF3, IRF7 and β-actin mRNA. ***P < 0.001 (HCECs *versus* HCECs/EBV). Data (A–C) are presented as the mean of three independent experiments, and error bars represent SDs of the means. (D) Western blots were performed for phospho-IRF3, IRF3, phospho-IRF7, IRF7 and β-actin protein. (E) Cytosolic extracts (left panel) or nuclear extracts (right panel) were analysed by western blot using Abs against phospho-IRF3, IRF3, phospho-IRF7 and IRF7. A nuclear marker, PARP, and cytosol marker, β-tubulin, were used to verify the purity of each fraction. Fractionation was performed as described in Materials and Methods. Results are representative of three independent experiments.

RIG-I and TLR3 also lead to the activation of several transcription factors, including IRF3 and IRF7 34. Pharmacological inhibition of TAK1 reduces IFN-β expression, and IRF3 is activated in TLR3-ligand stimulated IRAK1-deficient macrophages 36. To investigate the effect of EBERs on type I interferon production, the mRNA expression and protein levels of IFN-β and related proteins were examined in HCECs/EBV. mRNA expression and production of IFN-β were significantly induced in HCECs/EBV (Fig.3A and B). mRNA expression and phosphorylated IRF3 and IRF7 were increased in HCECs/EBV compared to non-infected HCECs (Fig.3C and D). Nuclear accumulation of phosphorylated IRF3 and IRF7 was also observed in HCECs/EBV (Fig.3E). These data suggest that the viral RNA components play important roles in the production of IFN-β through activation of IRF3 and IRF7 after binding to up-regulated TLR3 in corneal inflammation.

### Activation of IRFs and NF-κB is mainly regulated by both TLR3/TRIF and RIG-I/RIP-1 pathways in HCECs/EBV

**Figure 4:**
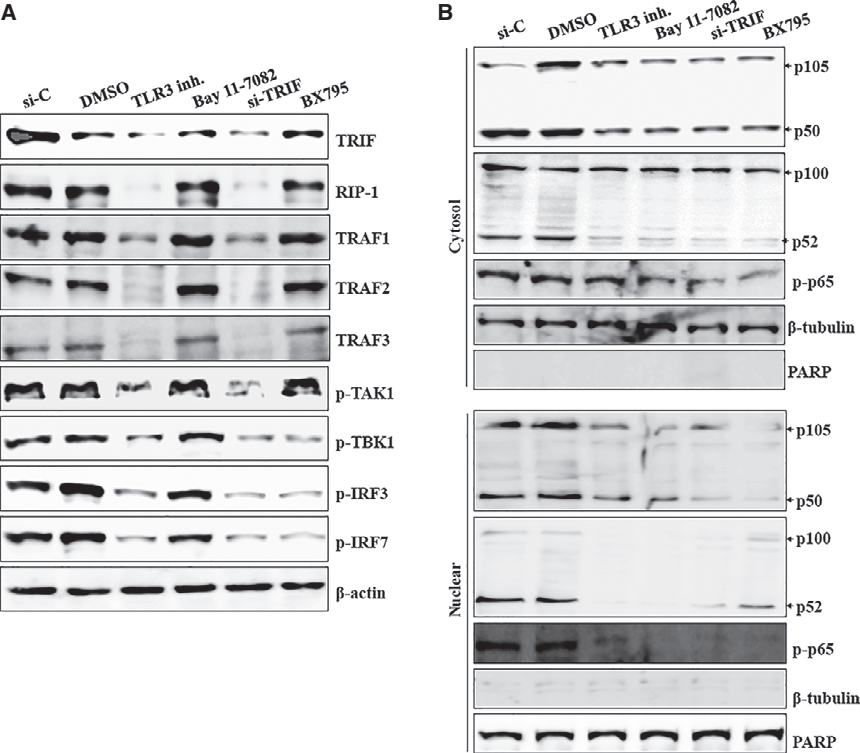
TLR3/TRIF regulates activation of IRFs and NF-κB through modulation of RIP-1 and TRAF family proteins in HCECs/EBV. (A) EBV-infected HCECs were treated with the indicated inhibitors and transfected with TRIF-siRNA (200 nM) or control-siRNA for 48 hrs prior to experiments. Different inhibitors were applied at the following concentrations: TLR3/dsRNA inhibitor (50 nM), NF-κB inhibitor Bay 11-7082 (5 μM), TBK1 inhibitor BX795 (10 nM). Total protein was subjected to Western blot analysis with the indicated antibodies. β-actin served as an internal control. (B) Cytosolic extracts (upper) or nuclear extracts (lower) were analysed by Western blot using the indicated antibodies. A nuclear marker, PARP, and cytosol marker, β-tubulin, were used to verify the purity of each fraction. Fractionation was performed as described in Materials and Methods. Results are representative of three independent experiments.

**Figure 5:**
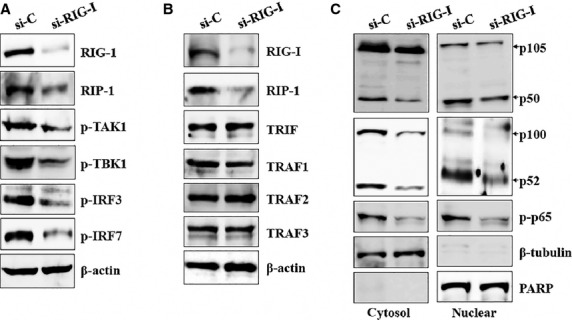
RIG-1 mainly regulates activation of TAK1/TBK1/IRFs pathway in HCECs/EBV. (A and B) EBV-infected HCECs were transfected with RIG-I-siRNA or control-siRNA for 48 hrs prior to experiments. Total protein was subjected to Western blot analysis with the indicated antibodies. β-actin served as an internal control. (C) Cytosolic extracts (left panel) or nuclear extracts (right panel) were analysed by Western blot using the indicated antibodies. A nuclear marker, PARP, and cytosol marker, β-tubulin, were used to verify the purity of each fraction. Fractionation was performed as described in Materials and Methods. Results are representative of three independent experiments.

Toll-like receptor-3-mediated signalling is divided into the NF-κB and IRF3 pathways at the level of TRIF 37. The N-terminus of TRIF interacts with TRAF6 and TBK1 38, whereas the C-terminus of TRIF binds RIP-1 39. RIG-I recruits RIP-1 and caspase-8 complex after viral infection. Caspase-8-mediated proteolytic processing of RIP-1 serves an antagonistic regulatory role on IRF3 after RNA virus infection 40. Inhibitors and siRNA transfection were used to examine which pathway or molecule mainly controls signal transduction in HCECs/EBV. First, we investigated the TLR3/TRIF pathway with TLR3 inhibitor or TRIF siRNA that led to decreased levels of RIP-1 and TRAF1-3 (Fig.4A). Reduced phosphorylation of TAK1, TBK1, IRF3, and IRF7 was also observed after blockade of TLR3 or TRIF signalling (Fig.4A and Fig. S3A). The activation and nuclear translocation of NF-κB was considerably reduced in HCECs/EBV after silencing TLR3 or TRIF signalling (Fig.4B). Despite the continuous expression of phosphorylated TAK1, TBK1, IRF3 and IRF7 protein, NF-κB inhibitor (Bay 11-7082) effectively inhibited NF-κB activation in both cytosol and nuclear fractions (Fig.4A and B). BX795, a TBK1/IKKε inhibitor, not only blocked NF-κB activation through blocking the phosphorylation of TBK1 but also inhibited the phosphorylation of IRF3 and IRF7, whereas phosphorylated TAK1 was maintained (Fig.4A and B). Next, we examined the RIG-I/RIP-1 signalling pathway. Although RIP-1, TAK1, TBK1, IRF3 and IRF7 activation were significantly reduced after transfection with RIG-I siRNA, the TRIF-TRAF family pathway was not affected by knocking down RIG-I (Fig.5A and B, Fig. S3B). Nuclear accumulation of active NF-κB subunits p50 and p52 or phosphorylation of p65 was also blocked after transfection with RIG-I siRNA (Fig.5C). These data suggest that the both TLR3/TRIF and RIG-I/RIP-1 pathways might be involved for NF-κB activation and affect the IRF3 pathway, which produces type I interferon in cornea epithelial after infection against viral RNA components.

### TLR3/TRIF/TBK1 pathway primarily triggers IL-32-mediated pro-inflammatory cytokines and IFN-β secretion in HCECs/EBV

**Figure 6:**
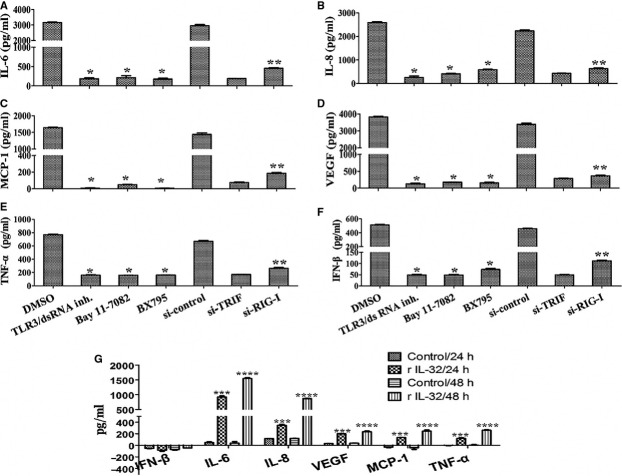
TLR3/TRIF pathway elicits IFN-β and IL-32-mediated pro-inflammatory cytokines secretion in HCECs/EBV. EBV-infected HCECs were treated with the indicated inhibitors and transfected with TRIF-siRNA, RIG-I-siRNA or control-siRNA for 48 hrs prior to experiments. Different inhibitors and siRNAs were applied at the following concentrations: TLR3/dsRNA inhibitor (50 nM), NF-κB inhibitor Bay 11-7082 (5 μM), TBK1 inhibitor BX795 (10 nM), TRIF-siRNA (200 nM) or RIG-I-siRNA (200 nM). After 48 hrs, culture supernatant was harvested and subjected to ELISA assay. Quantitative secretion levels of IL-6 (A), IL-8 (B), MCP-1 (C), VEGF (D), TNF-α (E) and IFN-β (F). Data are presented as the mean of three independent experiments, and error bars represent SDs of the means. Results are representative of three independent experiments. *P < 0.001 (DMSO control *versus* each inhibitor); **P < 0.01 (TRIF knockdown *versus* RIG-1 knockdown). (G) HCECs were cultured in the presence of recombinant IL-32γ (50 ng/ml) to confirm production of pro-inflammatory cytokines and IFN-β. Cell culture supernatants were collected at 24 and 48 hrs, and ELISA measured levels of various cytokines. Data are presented as the mean of three independent experiments, and error bars represent SDs of the means. ***P < 0.001(DMSO control *versus* recombinant IL-32γ treatment for 24 hrs); ****P < 0.001 (DMSO control *versus* recombinant IL-32γ treatment for 48 hrs).

We previously reported that pro-inflammatory cytokines, such as IL-6, IL-8, TNF-α, MCP-1 and VEGF, are produced in HCECs/EBV 31. Because NF-κB activation and IRF3 phosphorylation were effectively blocked by TLR3 inhibitor and TRIF siRNA, we next examined whether down-regulation of TLR3/TRIF or RIG-I has effect on production of pro-inflammatory cytokines and IFN-β. IL-32α and IL-32γ productions were inhibited in HCECs/EBV treated with several inhibitors and transfected with TRIF siRNA or RIG-I siRNA (Fig. S4A and B). Pro-inflammatory cytokines and IFN-β were not produced in HCECs/EBV after treatment with TLR3 inhibitors, NF-κB inhibitor and TBK inhibitor. Production of pro-inflammatory cytokines and IFN-β was also effectively blocked in HCECs/EBV transfected with TRIF siRNA and RIG-I siRNA. Meanwhile, in comparison with TRIF siRNA transfection, RIG-I siRNA transfection less effectively inhibited the production of pro-inflammatory cytokines and IFN-β in HCECs/EBV (Fig.6A–F). We next evaluated whether IL-32 stimulates the production of pro-inflammatory cytokines or IFN-β in HCECs after treatment with recombinant IL-32γ. Pro-inflammatory cytokines, including IL-6, IL-8, TNF-α, MCP-1 and VEGF, were secreted at 24 hrs in HCECs after stimulation with recombinant IL-32γ compared to non-stimulated HCECs. IFN-β was not detected after stimulation with recombinant IL-32γ (Fig.6G). These data suggest that IL-32 production through TLR3/TRIF/TBK signalling is a critical pathway for producing pro-inflammatory cytokines and IFN-β secretion through regulation of NF-κB activation in HCECs/EBV.

### RIP-1 controls signals from both TLR3/TRIF and RIG-I pathways in HCECs/EBV

**Figure 7:**
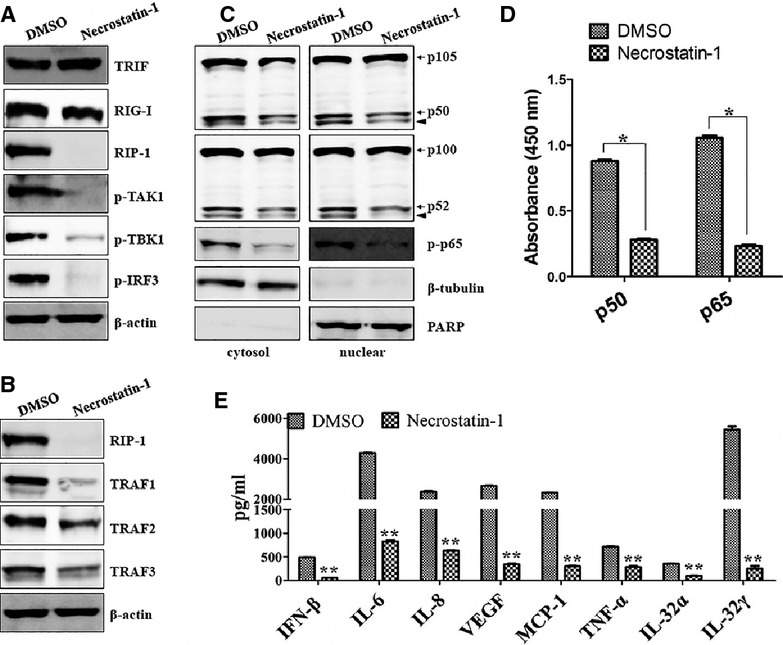
RIP-1 controls signals originating from the TLR3/TRIF and RIG-I pathways in HCECs/EBV. EBV-infected HCECs were treated with 30 μM of the RIP-1 inhibitor, necrostatin-1, for 48 hrs. (A and B) Total proteins were extracted from cell lysates and western blots for indicated proteins were performed. (C) Cytosolic extracts (left panel) or nuclear extracts (right panel) were examined by Western blot analysis using Abs against p105/p50, p100/p52 and phospho-p65. Solid arrowhead indicates a non-specific band. A nuclear marker, PARP, and cytosolic marker, β-tubulin, were used to assess the purity of each fraction. (D) ELISA measured NF-κB DNA-binding activity in nuclear extracts. Transcription factor NF-κB p50 combo and p65 combo (in Kit) served as positive controls of NF-κB activity. ELISA results are expressed as relative absorbance. Data represent mean ± SD of three independent experiments. *P < 0.01 (DMSO control *versus* Necrostatin-1). (E) After treatment with necrostatin-1 for 48 hrs, culture supernatant was harvested and subjected to ELISA assay. Quantitative secretion levels of IFN-β, IL-6, IL-8, VEGF, MCP-1, TNF-α, IL-32α and IL-32γ ELISA. **P < 0.001 (DMSO control *versus* Necrostatin-1). Data are presented as the mean of three independent experiments, and error bars represent SDs of the means. Results are representative of three independent experiments.

RIP-1 mediates signals from both TLR3 and RIG-I for activation of the NF-κB and IRF3 pathways 39,40. In a previous study, we observed that the expression of RIP-1 was decreased after blocking signals from TLR3 (Fig.4A) and RIG-I (Fig.5A) in HCECs/EBV. Next, we investigated whether RIP-1 controls signals from both TLR3 and RIG-I to regulate cytokine secretion in HCECs/EBV. RIG-I and TRIF expression were not affected by Necrostatin-1, a RIP-1 inhibitor, whereas Necrostatin-1 dramatically reduced the phosphorylation of RIP-1 target proteins, such as TAK1, TBK1, and IRF3 (Fig.7A). TRIF-related TRAF-family proteins also decreased, but not completely, after treatment with Necrostatin-1 in HCECs/EBV (Fig.7B). Despite the low expression of TRAF1, TRAF2 and TRAF3, phosphorylation of TAK1 and TBK1 completely blocked pharmacological inhibition of RIP-1 (Fig.7A and B). The activation and nuclear accumulation of active NF-κB subunits were suppressed by Necrostatin-1 (Fig.7C and D). Pro-inflammatory cytokines and IFN-β secretion were measured by ELISA after treatment with RIP-1 inhibitor. The IL-32α and γ isoforms were reduced and pro-inflammatory cytokines induced by IL-32, including IL-6, IL-8, TNF-α, VEGF and MCP-1, decreased significantly. A decrease in IFN-β production was also observed after treatment with Necrostatin-1 in HCECs/EBV (Fig.7E). These data suggest that RIP-1 plays a critical role in regulating NF-κB signals that produce pro-inflammatory cytokines and type I interferon in HCECs/EBV.

## Discussion

**Figure 8:**
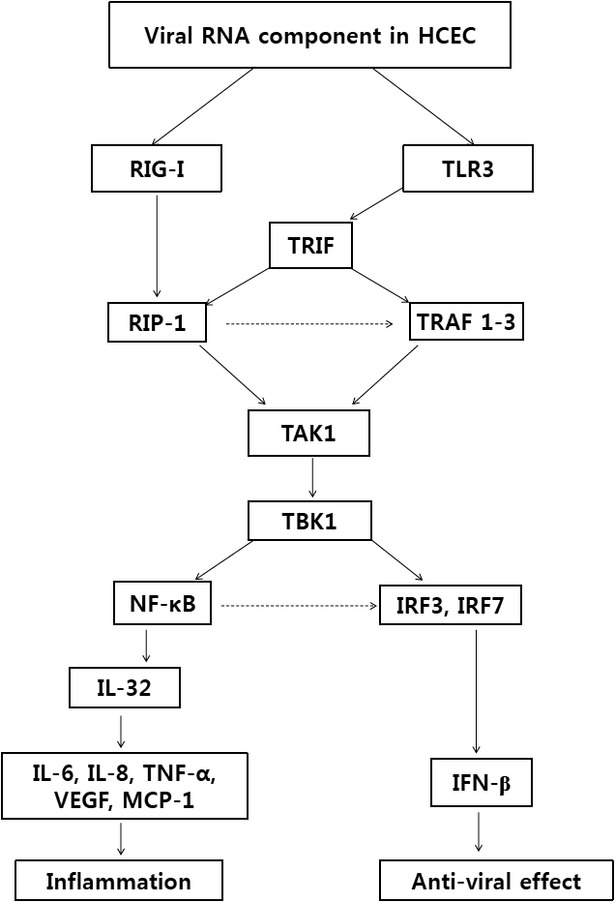
TLR3/TRIF mainly regulates IL-32-mediated pro-inflammatory cytokine and IFN-β secretion through activation of RIP-1 and TRAF family proteins. Corneal epithelium exposure to viral or microbial products, including double-stranded RNA (poly(I:C)), initiates TLR3 or RIG-I dependent signalling. In this study, both signalling pathways utilize RIP-1 and TRAF1-3, but not TRAF6. The TLR3/TRIF/RIP-1 pathway activates NF-κB, resulting in expression of IL-32-mediated pro-inflammatory cytokines. This pathway also activates transcription factors IRF-3 and IRF-7 to produce IFN-β through NF-κB activation. These observations extend our understanding of TLR/TRIF signalling by demonstrating the function of IL-32 secretion as a mediator of inflammation in corneal epithelial cells.

Toll-like receptor-3 activation of epithelial cells on corneal surfaces is closely controlled to minimize responses that might damage the eye and cause visual impairment. The inflammatory response to a variety of insults includes the production of various cytokines and chemokines. However, it is still unclear how the TLR3 signalling pathway and production of related pro-inflammatory cytokines are regulated in the ocular surface mucosa. Human studies have demonstrated alterations in the cytokine protein expression profiles on the ocular surfaces of patients with keratoconjunctivitis sicca, one of the most common problems of patients at ophthalmology clinics. These include increased concentrations of IL-1α, IL-6, IL-8, IL-12 and TNF-α 41,42. Viral activation of TLR3- and RIG-I-mediated pathways can be simulated by the addition or transfection of synthetic dsRNA (poly(I:C)) into cells 43. However, we cannot verify clearly how the real viruses work to induce immune responses inside cell. Using HCECs/EBV as a model of human viral keratoconjunctivitis, this study showed that the TLR3/TRIF pathway preferentially controls IL-32-mediated pro-inflammatory cytokines and IFN-β secretion through regulation of RIP-1 and TRAF-family proteins (Fig.8).

Although viral RNA components presumably bind to TLR3 or RIG-I to induce pro-inflammatory cytokines or anti-viral interferon, conditions that preferentially respond with TLR3 or RIG-I remain unclear. Phosphorylation of specific tyrosine residues in the cytoplasmic domain of TLR3 leads to recruitment of an adaptor protein TRIF, which recruits the TRIF/TBK1/IRF3 pathway or the TRIF/TRAF6/IKKαβ/NF-κB pathway 44. TRIF interaction with TBK1 is necessary for activation of IRF3, a transcription factor involved in the production of IFN-β 45. TLR3 can also activate NF-κB by the interaction of TRIF with RIP-1, leading to up-regulated production and secretion of other pro-inflammatory cytokines 3,46. In mammalian cells, RIG-I recognizes viral dsRNA and initiates signalling cascades that lead to activation of protein kinases IKKαβ, TBK1 and IKKε along with subsequent activation of transcription factors NF-κB and IRF3 47. TAK1 has also been reported to be involved in signalling TRAF6 to activate NF-κB 48. However, the relationship between RIP-1 and the TRIF/TRAF1, TRAF2, and TRAF3 pathways to produce cytokines after viral infection is unknown. In this study, we observed the TLR3/TRIF/RIP-1 pathway also involved in TRAF1, TRAF2 and TRAF3 activation, but not TRAF6 after stimulation by dsRNA or viral infection in the corneal epithelium. Subsequent TAK1 activation responded to TLR or RIG-I activation to produce cytokines. Despite the expression of TRAF1, TRAF2 and TRAF3, phosphorylation of TAK1 completely blocked pharmacological inhibition of RIP-1. These results indicate that RIP-1 activation might be preferentially required for TAK1 activation regardless of TRAF activation.

Interferon-beta promoter stimulator-1 (IPS-1), the TRIF equivalent in the RIG-I pathway, links RIG-I to the IKKε/TBK1/IRF3 and TRAF6/FADD/NF-κB pathways 34. In the NF-κB branch, FADD and RIP-1 may be conduits between IPS-1 and IKKαβ 34. Although the IPS-1 interaction with TRAF6 provides another link to IKKαβ, the linkage of RIG-I or RIP-1 to TBK1 and NF-κB for production of pro-inflammatory cytokines and anti-viral IFN-β is unclear in viral infections 49. Pharmacological inhibition of RIP-1 with Necrostatin-1 blocked activation of both the TRAFs/TAK/NF-κB pathway and the TBK1/IRF3 pathway in this study. The data suggest that TRAFs relay signals to the TBK1/IKKε/NF-κB pathway under the partial influence of RIP-1, which might control NF-κB-mediated IL-32 production to induce various pro-inflammatory cytokines in HCECs/EBV. IL-32 stimulates and differentiates monocytes into macrophage. The differentiated macrophages are potent producers of key inflammatory cytokines such as TNFα, IL-1β and IL-6 in inflammatory bowel disease 50,51. IL-32γ expression in fibroblast-like synoviocytes induces the production of IL-1β, IL-6 and IL-8 52. From these results, our data also suggest that the modulation of IL-32 through regulation of TLR3/TRIF and RIG-I pathways might be one of the major targets in inflammatory and/or autoimmune diseases, including ocular surface mucosa diseases.

Matthys and colleagues recently reported that Gn proteins from Hantavirus, which harbour elements in their 142-residue cytoplasmic tails, regulate RIG-I/TBK1 pathway-directed IRF3 phosphorylation and IFN-β induction 53. In this study, knock down of TRIF by siRNA completely blocked the RIP-1/TAK1/TBK1/IRF3 pathway in HCECs/EBV. RIG-I silencing by siRNA affected the TBK1/IRF3 signal pathway, but not the TRIF/TRAFs pathway. We also observed that production of pro-inflammatory cytokines and IFN-β was less effectively inhibited after transfection of RIG-I siRNA in HCECs/EBV. These results indicate that the TLR3/TRIF pathway is the main signalling mechanism to induce production of inflammatory cytokines and IFN-β in corneal epithelia after viral infection through regulation of RIP-1 and TRAFs.

The cornea epithelium serves to maintain corneal transparency. The formation of TJs between adjacent epithelial cells at the apical plasma membrane 54,55 provides physical and functional barriers 56. TNF-α disrupts the barrier function of cultured human corneal epithelial cells 57. Zonula occludens (ZO-1, ZO-2 and ZO-3), occludin, claudin and AJs (β-catenin, p120 catenin) are thought to determine corneal barrier function 58–60. Inflammation induced by TNF-α can damage the barrier and transparency functions of the cornea to cause vision changes. HCECs/EBV up-regulate the expression of pro-inflammatory cytokines, including TNF-α, IL-6 and IL-8. These cells not only lose the expression of E-cadherin, β-catenin and ZO-1 but increase expression of mesenchymal markers such as N-cadherin and Vimentin. For this reason, the HCECs/EBV cell line was chosen as a research model for viral keratoconjunctivitis to investigate the regulatory mechanism of IL-32-mediated pro-inflammatory cytokine secretion.

Interleukin-32 lacks sequence homology with presently known cytokine families. IL-32 is a strong inducer of pro-inflammatory cytokines such as TNF-α, IL-1β, IL-6 and IL-8 17. There are six isoforms (α, β, γ, δ, ε and ξ) caused by alternative mRNA splicing, resulting in proteins with molecular weights ranging from 14.9 to 26.7 kD 14,15. All isoforms are biologically active, and the recombinant IL-32γ isoform showed the highest biological activity 61. Although functional differences between these isoforms remain unknown, the significant role of IL-32 has been reported in the development of several diseases, including arthritis, psoriasis, ulcerative colitis and Crohn's disease 62. IL-32 also modulates signals induced by specific TLRs and nucleotide oligomerization domain (NOD) ligands. IL-32 synergized with NOD1 and NOD2 ligands for the synthesis of IL-1β and IL-6 *via* activation of caspase-1 18. IL-32 expression is greatly increased in HCECs/EBV and poly(I:C)-stimulated HCECs. Pro-inflammatory cytokines, including TNF-α, IL-1β, IL-6 and IL-8, were significantly down-regulated after blocking the TLR3-related signalling pathway with inhibitors or siRNA compared to group of RIG-1 silencing. Although TLR3/TRIF/RIP-1/TBK1 was found to be a main pathway for producing pro-inflammatory cytokines and IFN-β, RIG-1 also affected IL-32 and IFN-β secretion. However, further studies are needed to verify the effects of RIG-1 on IL-32 and pro-inflammatory cytokine secretion. Activation of TBK1 and IKKε along with subsequent activation of IRF3 and IRF7 mainly leads to the induction of IFN-β 63,64. IFN gene induction also requires NF-κB, which cooperates with IRF3 and IRF7 to assemble the IFN-β promoter and recruit co-activators and chromatin-remodelling factors 65. Despite phosphorylation and activation of TBK1, IRF3 and IRF7, IFN-β secretion was inhibited after treatment with Bay-11-7082, a NF-κB inhibitor. This result is consistent with the theory that a non-canonical NF-κB pathway regulates production of IFN-β 66. The Phosphorylation of IRF3 and secretion of IFN-β were inhibited by gene silencing of TRIF and RIG-1 with siRNA or pharmacological inhibition of NF-κB as IL-32 secretion was blocked. However, IFN-β production was not induced after treatment with recombinant IL-32. These results suggest that NF-κB activation has influence on the secretion of IL-32 and IFN-β. However, signalling pathway for production of IFN-β is separated at downstream of NF-κB (Fig.8).

Our results reveal that TRIF-dependent RIP-1 and TRAFs activation by TLR3 is critical for IL-32-mediated pro-inflammatory cytokines production in ocular mucous surfaces after viral infection. RIP-1 controls signalling from both TLR3/TRIF and RIG-I pathways. These results suggest that appropriate regulation of the TRIF/RIP-1 pathway and subsequent NF-κB activation may decrease severe corneal damage in viral infections by inhibiting pro-inflammatory cytokines.

## Supporting Information

**Figure S1** (A) mRNA levels of EBER1 and EBER2 expression in EBV-infected and uninfected HCECs measured using real-time PCR. **Figure S2** Total RNA was extracted from cell for real-time PCR. **Figure S3** (A and B) EBV-infected HCECs were treated with the indicated inhibitors and transfected with TRIF siRNA, RIG-I-siRNA or control-siRNA for 48 h prior to experiments. **Figure S4** Quantitative secretion levels of IL-32α (A) and IL-32γ (B).
