# Supplementary material for: TLR3/TRIF signalling pathway regulates IL-32 and IFN-β secretion through activation of RIP-1 and TRAF in the human cornea
Source: J Cell Mol Med. 2015 Mar 6;19(5):1042–54. doi: 10.1111/jcmm.12495 (PMC4420606; doi:10.1111/jcmm.12495)
Supplement: Supplementary file 1 [file jcmm0019-1042-sd1.pdf]

Supplementary Fig. 1

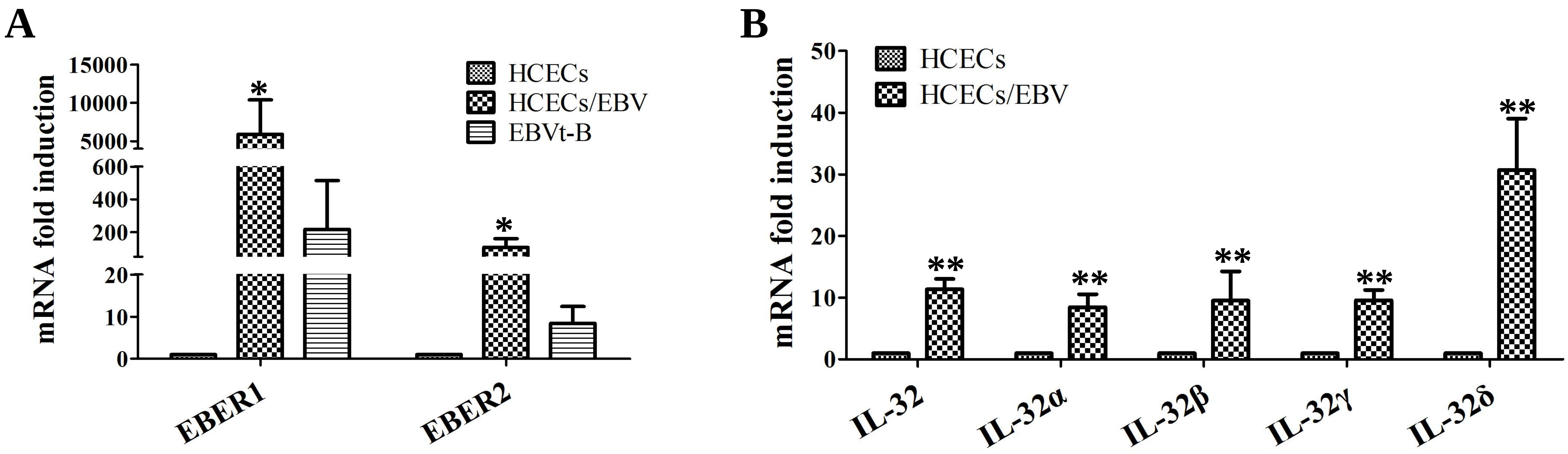

SUPPLEMENTAL FIGURE 1. (**A**) mRNA levels of EBER1 and EBER2 expression in EBV-infected and uninfected HCECs measured using real-time PCR. EBV-transformed B cells were used as a positive control. *, p<0.001 (HCECs versus HCECs/EBV). **(B)** Real-time PCR for total IL-32, IL-32α, IL-32β, IL-32γ and IL-32δ. **, p<0.001 (HCECs versus HCECs/EBV). Column indicates mRNA fold induction of EBV-infected HCECs compared to HCECs. Results were normalized using β-actin as control.

Supplementary Fig. 2

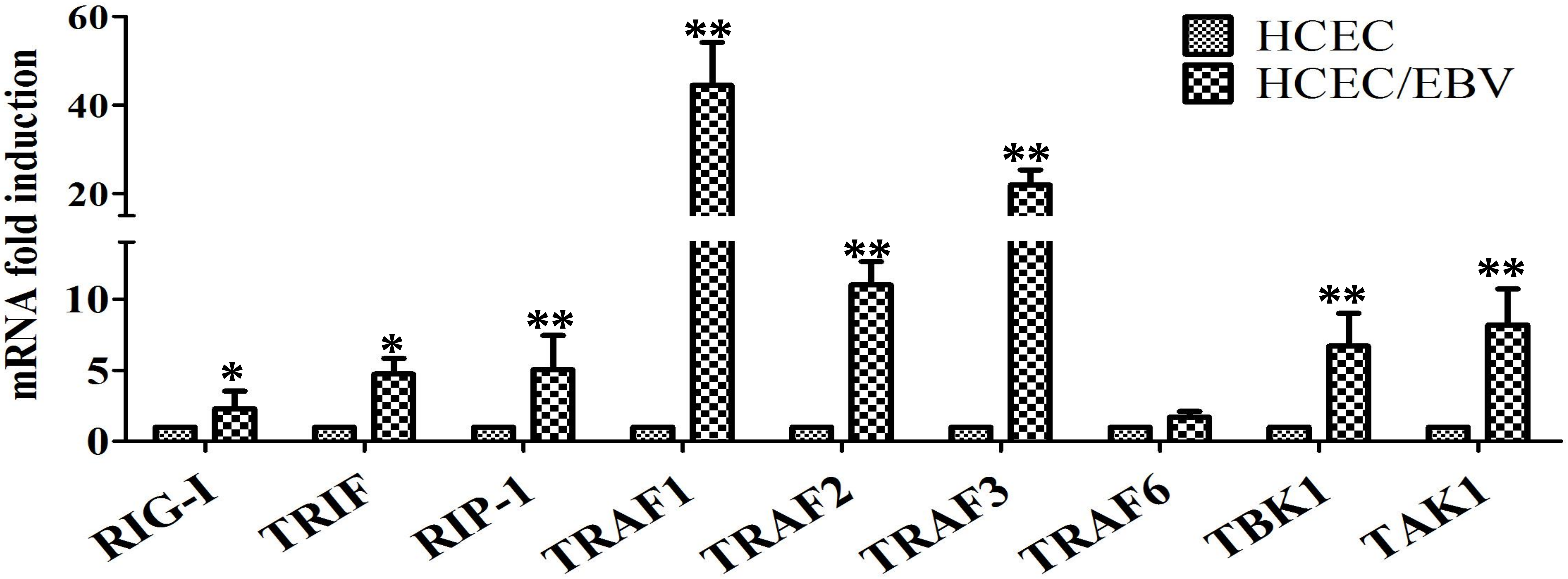

SUPPLEMENTAL FIGURE 2. Total RNA was extracted from cell for real-time PCR. Compare the mRNA level of RIG-I, TRIF, RIP-1, TRAF1, TRAF2, TRAF3, TRAF6, TBK1, TAK1 in EBV-infected and uninfected HCECs. β-actin was used as control. *, p<0.01 (HCECs versus HCECs/EBV); **, p<0.001 (HCECs versus HCECs/EBV). Data are presented as the mean of three independent experiments, and error bars represent SDs of the means. Results are representative of three independent experiments.

Supplementary Fig. 3

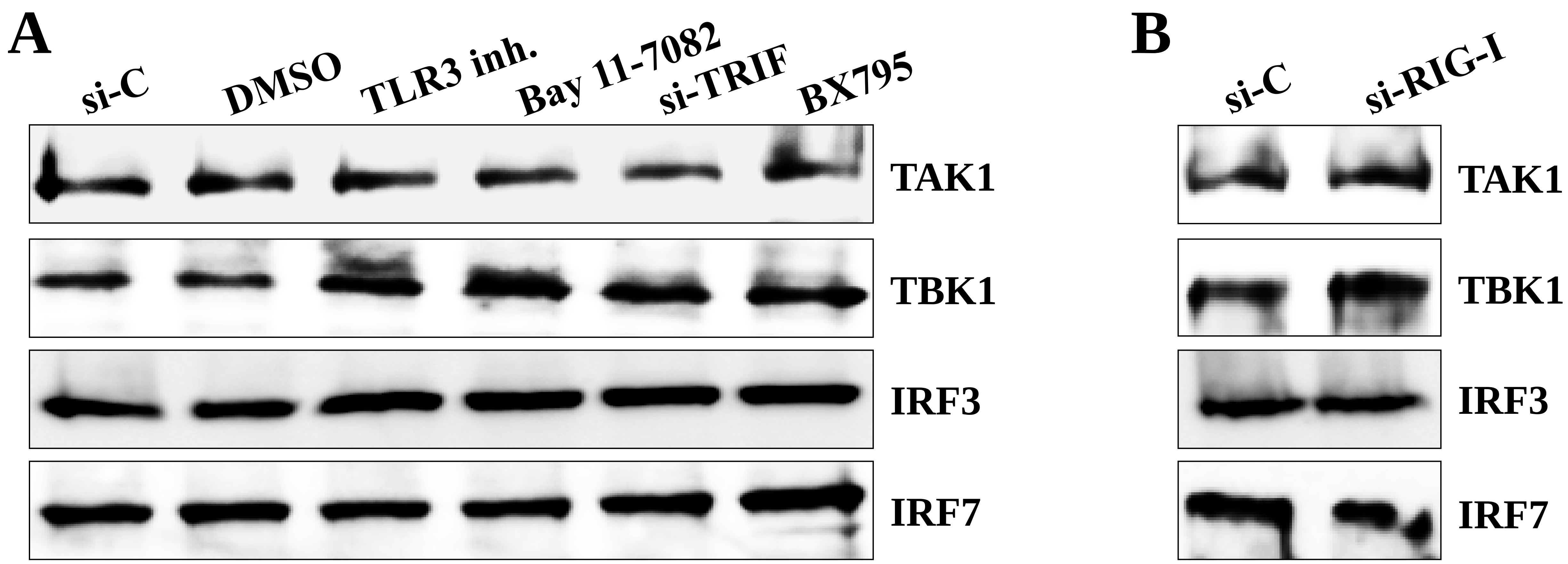

SUPPLEMENTAL FIGURE 3. (**A and B**) EBV-infected HCECs were treated with the indicated inhibitors and transfected with TRIF-siRNA, RIG-I-siRNA or control-siRNA for 48 h prior to experiments. Different inhibitors and siRNAs were applied at the following concentrations: TLR3/dsRNA inhibitor (50 nM), NF-κB inhibitor Bay 11-7082 (5 μM), TBK1 inhibitor BX795 (10 nM), TRIF-siRNA (200 nM) or RIG-I-siRNA (200 nM). Total protein was subjected to Western blot analysis with the indicated antibodies. β-actin served as an internal control. Results are representative of three independent experiments.

Supplementary Fig. 4

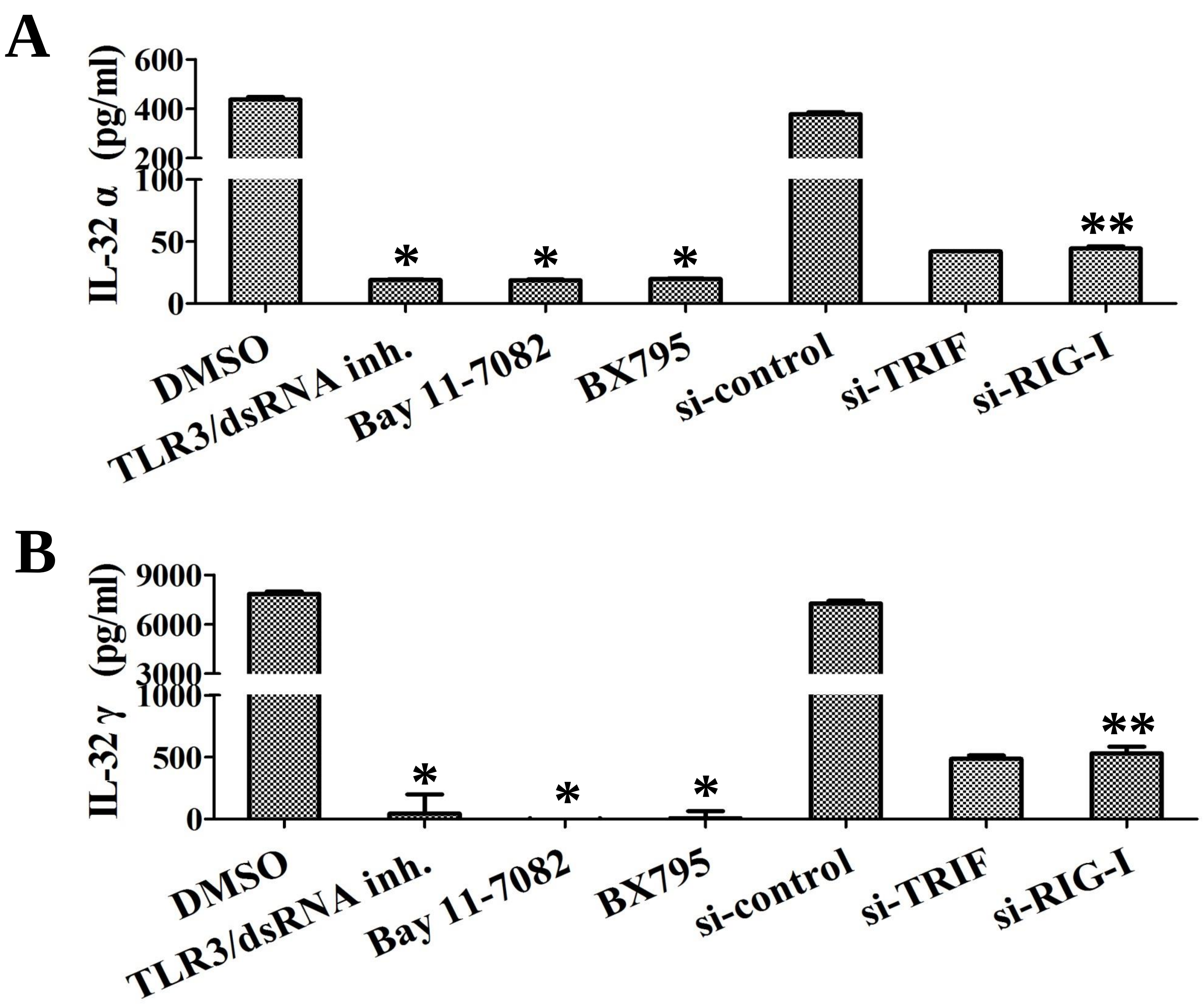

SUPPLEMENTAL FIGURE 4. Quantitative secretion levels of IL-32α (**A**) and IL-32γ (**B**). Different inhibitors and siRNAs were applied at the following concentrations: TLR3/dsRNA inhibitor (50 nM), NF-κB inhibitor Bay 11-7082 (5 μM), TBK1 inhibitor BX795 (10 nM), TRIF-siRNA (200 nM) or RIG-I-siRNA (200 nM). After 48 h, culture supernatant was harvested and subjected to ELISA assay. *, $p<0.001$ (DMSO control versus each inhibitor); **, $p< 0.05$ (TRIF knockdown versus RIG-1 knockdown). Data are presented as the mean of three independent experiments, and error bars represent SDs of the means. Results are representative of three independent experiments.
